# Serological diagnosis of *Toxoplasma gondii*: analysis of false-positive IgG results and implications

**DOI:** 10.1051/parasite/2020006

**Published:** 2020-02-07

**Authors:** Loïc Simon, Judith Fillaux, Aurélie Guigon, Rose-Anne Lavergne, Odile Villard, Isabelle Villena, Pierre Marty, Christelle Pomares

**Affiliations:** 1 Service de Parasitologie-Mycologie, CHU de Nice, Université Côte d’Azur 06202 Nice France; 2 Inserm U1065, C3M 06204 Nice France; 3 Service de Parasitologie-Mycologie, CHU de Toulouse 31300 Toulouse France; 4 PharmaDev, IRD UMR 152, Université de Toulouse 31062 Toulouse France; 5 Service de Microbiologie, Hôpital La Source, CHR d’Orléans 45100 Orléans France; 6 Parasitologie-Mycologie, CHU de Nantes 44093 Nantes France; 7 Université de Nantes, Nantes Atlantique Universités, EA1155-IICiMed, Institut de Recherche en Santé 2 44200 Nantes France; 8 Institut de Parasitologie et de Pathologie Tropicale, EA 7292, Fédération de Médecine Translationnelle, Université de Strasbourg 67000 Strasbourg France; 9 EA7510, ESCAPE, Laboratoire de Parasitologie-Mycologie, Université de Reims Champagne-Ardenne, SFR Cap Santé FED 4231 51096 Reims France

**Keywords:** *Toxoplasma gondii*, IgG, False-positive, Serology, Architect, Toxoplasmosis

## Abstract

*Background*: Primary infection by *Toxoplasma gondii* in pregnant women can result in serious outcomes for the foetus. A false-positive IgG result during pregnancy can lead to a misdiagnosis of past infection and to stopping preventive measures. We collected 189 sera with positive Architect^®^ Toxo IgG assay (Abbott Laboratories) and negative IgG results with at least two other serological tests, in order to find an explanation for the suspected false-positive IgG results. We used the *recom*Line Toxoplasma IgG^®^ immunoblot (Mikrogen Diagnostik) to search for specific antigenic reactivities of the sera, and the LDBio Toxo II IgG^®^ immunoblot (LDBio Diagnostics) as a confirmatory test. *Results*: The bands GRA8 and/or GRA7 were positive for 148 samples (78.3%). GRA8 was the most frequent band, appearing in 133 patterns (70.4%), whereas GRA7 was present for 49 samples (25.9%). Of the 81 samples tested with LDBio^®^, 23 (28.4%) turned out to be positive. Of the 58 negative LDBio^®^ tests (71.6%) (real false-positive Architect^®^ IgG), 23 samples (39.6%) did not show either a GRA8 or p30 band by *recom*Line^®^. Their false positivity with Architect^®^ remains unexplained since Abbott uses these two recombinant antigens for their assay. *Conclusions*: The Architect^®^ IgG false positivity for *T. gondii* seems to be due to reactivity against GRA8 for the majority of the sera and GRA7 to a lesser extent. The hypothesis of past contact with parasites genetically close to *T. gondii* such as *Hammondia hammondi* or *Neospora caninum* seems promising and should be assessed further.

## Abstract (fr)

*Contexte* : La primo-infection à *Toxoplasma gondii* chez la femme enceinte peut avoir de graves conséquences pour le fœtus. Un résultat IgG faussement positif pendant la grossesse peut mener à un diagnostic erroné d’infection ancienne et à stopper les mesures préventives. Nous avons collecté 189 sérums présentant un résultat Architect^®^ Toxo IgG (Abbott Laboratories) positif ainsi qu’un résultat IgG négatif par au moins deux autres tests sérologiques, dans le but de trouver une explication aux résultats IgG suspectés faux positifs. Nous avons utilisé l’immunoblot *recom*Line Toxoplasma IgG^®^ (Mikrogen Diagnostik) pour chercher certaines réactivités antigéniques spécifiques des sérums et l’immunoblot LDBio Toxo II IgG^®^ (LDBio Diagnostics) comme test de confirmation. *Résultats* : Les bandes GRA8 et/ou GRA7 étaient positives pour 148 (78,3 %) échantillons. GRA8 était la bande la plus fréquente, apparaissant dans 133 (70,4 %) profils alors que GRA7 était présente pour 49 (25,9 %) échantillons. Sur les 81 échantillons testés en LDBio^®^, 23 (28,4 %) se sont révélés positifs. Sur les 58 (71,6 %) tests LDBio^®^ négatifs (réels faux positifs IgG Architect^®^), 23 (39,6 %) échantillons n’ont montré ni bande GRA8 ni bande p30 en *recom*Line^®^ et leur fausse positivité reste donc inexpliquée puisque Abbott utilise ces deux antigènes recombinants dans son test. *Conclusions* : La fausse positivité IgG Architect^®^ pour *T. gondii* semble être due à une réactivité envers la protéine GRA8 pour la majorité des sérums et envers GRA7 dans une moindre mesure. L’hypothèse d’un contact passé avec des parasites génétiquement proches de *T. gondii* comme *Hammondia hammondi* ou *Neospora caninum* semble prometteuse et devrait être approfondie.

## Introduction

Toxoplasmosis is a parasitic disease due to *Toxoplasma gondii*, an obligate intracellular protozoan with a worldwide distribution. The life cycle of the parasite involves sexual reproduction in definitive hosts from the Felidae family, and a broad range of intermediate hosts including mammals and birds [18, 26, 30, 41, 46, 47]. Human infection occurs mainly by ingestion of *T. gondii* oocysts present on raw and unwashed vegetables, and through consumption of raw or undercooked meat containing cysts of the parasite [6, 22, 48]. Although *T. gondii* primary infection is usually asymptomatic in the healthy population, it can be life-threatening for others, like immunocompromised patients. In these hosts, acute infection or reactivation of a past infection can lead to severe and possibly lethal diseases (cerebral, pulmonary, or disseminated toxoplasmosis) [25, 27, 40]. In particular, primary infection in pregnant women and reactivation in immunocompromised pregnant women can be the cause of congenital toxoplasmosis with the risk of serious outcomes for the foetus, mainly retinitis pigmentosa, hydrocephaly, or even death *in utero* [4, 24, 44].

In some countries, the health authorities have set up a prenatal screening program for *T. gondii* [33, 34, 43]. In France, pregnant women are tested in the early weeks of pregnancy for the presence of specific IgG and IgM against *T. gondii*. The presence of *T. gondii* IgG at a stable level without IgM is in favour of a past infection. These women are considered to be immunised against *T. gondii* and follow-up is no longer performed. In seronegative pregnant women, monthly screening will be performed to allow early diagnosis and treatment of an acute infection in order to prevent transplacental transmission of the parasite to the foetus [43]. This highlights the importance of an accurate and reliable test for the detection of specific IgG, given that a false-positive result can lead to a misdiagnosis of past infection, and to stopping surveillance and preventive measures in a pregnant woman.

Currently, many serological tests are available for IgG detection with different sensitivities and specificities. Studies on the Architect^®^ Toxo IgG assay (Abbott Laboratories, North Chicago, IL, USA) show specificities and sensitivities ranging from 99.1% to 99.8% and 92.1% to 99.7%, respectively [17, 29, 32, 37, 42]. This assay is based on the principle of chemiluminescent microparticle immunoassay (CMIA). According to the manufacturer, Architect^®^ uses two *T. gondii* recombinant antigens for the immunoassay: a membrane protein of 30 kDa (= p30) called SAG1 (surface antigen 1), only found in the tachyzoite stage of the parasite; and a cytoplasmic protein of 35 kDa (= p35) called GRA8 (dense granule), found in the tachyzoite and bradyzoite stages of the parasite. Similarly, studies on other commonly used automated or semi-automated immunoassays (Advia Centaur^®^, AxSym^®^, Elecsys^®^, Enzygnost^®^, Liaison^®^, Platelia^®^, Vidas^®^, and Vidia^®^) show various specificities from 99.3% to 100%, while sensitivities range from 93.8% to 100% [32, 42]. Since the specificity is not 100% for some of these assays, pregnant women are exposed to the risk of being misdiagnosed as immunised, whereas they are not. In our article, we will focus on Architect^®^ in order to find a rational explanation for the suspected false-positive Architect^®^ Toxo IgG results. To our knowledge, this is the first study that tries to provide an explanation for discordant *T. gondii* IgG test results.

## Materials and methods

### Sample collection

The laboratory of Parasitology-Mycology at the University Hospital of Nice, France (Nice laboratory) is a member of the National Reference Centre for toxoplasmosis (Reims, France). This laboratory regularly receives human serum samples from other laboratories throughout France, in order to provide expertise for serological diagnosis of toxoplasmosis. From July 2009 to April 2018, sera from other laboratories with discordant *T. gondii* IgG results between different serological assays were sent to the Nice laboratory. The samples included in this study came from 21 cities in mainland France (Angers, Antibes, Arras, Embrun, Fréjus, Grenoble, Lisieux, Miramas, Nantes, Nice, Orléans, Reims, Saint-Denis, Saint-Etienne-du-Rouvray, Saint-Laurent-du-Var, Salon-de-Provence, Strasbourg, Toulon, and Toulouse), from the island of Corsica (Ajaccio), and French Guiana in South America (Saint-Laurent-du-Maroni); in addition to the samples from the Nice laboratory (Table 1).

**Table 1:** Origin of the samples.

City	Number of sera
Private laboratories	
Embrun	2
Lisieux	2
Miramas	1
Saint-Etienne-du-Rouvray	2
Saint-Laurent-du-Var	1
Salon-de-Provence	1
Hospital laboratories	
Ajaccio (Corsica)	3
Angers	4
Antibes	1
Arras	4
Fréjus	4
Grenoble	3
Nantes	30
Nice	60
Orléans	10
Reims	4
Saint-Denis	3
Saint-Laurent-du-Maroni (French Guiana)	3
Strasbourg	5
Toulon	1
Toulouse	45
Total	189

### Study design

Sera with positive Architect^®^ Toxo IgG test result and negative test result found by at least one other IgG assay were selected for further analyses. According to the manufacturer’s instructions, the Architect^®^ Toxo IgG assay is considered positive when the titre is ≥3 IU/mL. The other serological test results came from the following assays: Platelia^®^ Toxo IgG (Bio-Rad, Hercules, CA, USA), AxSym^®^ Toxo IgG (Abbott Laboratories), Toxolatex Fumouze^®^ (Biosynex, Eckbolsheim, France), Pastorex™ Toxo (Bio-Rad), Modified Agglutination Test (MAT), Toxo-Spot IF^®^ (bioMérieux, Marcy-l’Étoile, France), and Vidas^®^ Toxo IgG II (bioMérieux). In case only one of these assays was performed, the Nice laboratory performed a Vidas^®^ Toxo IgG II analysis. When a Vidas^®^ test result was the only one available, a Toxo-Screen DA^®^ (bioMérieux) was performed. For sera from the Nice laboratory, the Vidas^®^ and Toxo-Screen DA^®^ assays were always performed as part of the routine procedure of the laboratory. We grouped all these eight assays under the name “alternative tests”. Thus, each serum was positive by Architect^®^ Toxo IgG and negative by at least two assays among the alternative tests. Additionally, Architect^®^ Toxo IgM (Abbott Laboratories) was performed for all the sera in the study, and test results were all negative (index <0.6 according to the manufacturer’s instructions), except for one individual (Architect^®^ IgM index = 1.8) whose positive IgM were later confirmed on another sample as non-specific IgM.

To increase the power of the study, grey-zone IgG results by Architect^®^ were considered negative and were not included. Concerning the other three quantitative tests performed (Vidas^®^, Platelia^®^, and AxSym^®^), all IgG results obtained were below the grey-zone.

### Study population

In total, 189 samples collected from 176 individuals presenting positive IgG Architect^®^ (≥3 IU/mL) and negative IgG with at least two other serological tests were included in the study over a period of 9 years. The study population included 155 females (88.1%) and 21 males (11.9%). The mean age was 31.5 years, ranging from 1 to 87 years (including six children under 17 years).

### RecomLine^®^ immunoblot

The *recom*Line Toxoplasma IgG^®^ immunoblot (Mikrogen Diagnostik, Neuried, Germany) was performed on the 189 sera. This immunoblot is able to discriminate antibodies against the recombinant proteins of *T. gondii* ROP1c (= p66), GRA1 (= p24), GRA7 (= p29), GRA8 (= p35), SAG1 (= p30), MAG1 (= p65, p68), and MIC3. An additional antigen called rSAG1 (= p30, low concentration) is loaded to the strip as a marker of past infection. We hijacked the initial purpose of this diagnostic test and used it solely to elaborate a pattern of antibody reactivity, by noting the positive bands among the eight recombinant antigens coated, for each serum tested.

### LDBio^®^ immunoblot

Whenever possible, the LDBio Toxo II IgG^®^ immunoblot (LDBio Diagnostics, Lyon, France) was performed on the samples. We used this assay as a confirmatory test of the absence or presence of specific anti-*T. gondii* IgG [14]. According to the manufacturer, a positive LDBio^®^ immunoblot is defined by three apparent bands including p30 among the five *T. gondii* natural antigens coated, of molecular weights 30 kDa (= p30), 31 kDa, 33 kDa, 40 kDa, and 45 kDa. This assay was used to conclude whether the positive Architect^®^ Toxo IgG test results were true- or false-positives.

### Protein BLAST analysis (Basic Local Alignment Search Tool)

Protein sequences were blasted with NCBI’s online alignment tool using substitution matrix BLOSUM62. Similarity scores are expressed in bits, and Expect values (*E*-values) ≤ 10e–10 demonstrate significant homologies.

## Results

### Serological tests results

Across the 189 Architect^®^ Toxo IgG performed, the IgG values ranged from 3.0 to 235.1 IU/mL; the median was 5.6 IU/mL with an interquartile range of 6.5 IU/mL. Among the alternative tests, 157 (83.1%) Toxo-screen^®^ and 134 (70.9%) Vidas^®^ were performed. All the other alternative tests performed are detailed in Table 2.

**Table 2:** Diagnostic tests performed.

Analysis	Number of sera	Negative (%)	Positive (%)
Architect^®^ Toxo IgG	189	0 (0)	189 (100)
Toxo-Screen DA^®^	157	157 (100)	0 (0)
Vidas^®^ Toxo IgG	134	134 (100)	0 (0)
Platelia^®^ Toxo IgG	63	63 (100)	0 (0)
AxSYM^®^ Toxo IgG	21	21 (100)	0 (0)
Toxolatex Fumouze^®^	10	10 (100)	0 (0)
Toxo-Spot IF^®^	8	8 (100)	0 (0)
Pastorex™ Toxo	7	7 (100)	0 (0)
Modified Agglutination Test	4	4 (100)	0 (0)
LDBio-Toxo II IgG^®^	81	58 (71.6)	23 (28.4)

### RecomLine^®^ immunoblot results

**Figure 1:**
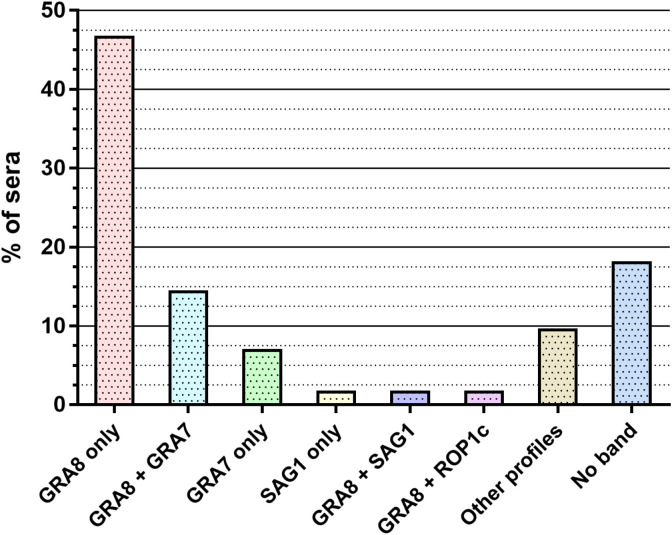
Description of the *recom*Line^®^ immunoblot profiles performed on the 189 suspected false-positive sera with Architect^®^.

We carried out 189 *recom*Line^®^ tests in total and analysed the pattern of positive bands among the eight recombinant antigens for each serum (Fig. 1). In our full sample set, we found 20 different profiles in which zero to maximum three bands per immunoblot were found. The majority of the samples were only positive for the GRA8 band (46.6%) but other frequent patterns were found, especially GRA8 + GRA7 (14.3%) and GRA7 only (6.9%). The remaining patterns included: SAG1 only (1.6%), GRA8 + SAG1 (1.6%), GRA8 + ROP1c (1.6%), ROP1c only (1.1%), GRA8 + SAG1 + ROP1c (1.1%), GRA8 + GRA7 + GRA1 (1.1%), GRA8 + GRA7 + ROP1c (1.1%), GRA8 + GRA7 + SAG1 (1.1%), MIC3 only (0.5%), GRA1 only (0.5%), GRA8 + GRA1 (0.5%), GRA8 + MAG1 (0.5%), GRA8 + MIC3 (0.5%), GRA7 + GRA1 (0.5%), GRA8 + GRA7 + MAG1 (0.5%), and GRA7 + GRA1 + ROP1c (0.4%). In 18% of the samples, the immunoblots did not show any positive band.

Taking into account all the patterns, we found GRA8 and/or GRA7 bands for 148 samples (78.3%). GRA8 was the most frequent band, appearing in total in 133 patterns (70.4%), whereas GRA7 was present for 49 samples (25.9%). We did not observe any specific pattern according to age or gender categories.

### LDBio^®^ immunoblot results

We were able to perform 81 LDBio^®^ tests on the leftover serum. Of these 81 samples tested, 23 (28.4%) turned out to be positive despite their 2–4 negative alternative tests (Table 2 and Supplementary Table 1). Most of the positive samples had similar profiles with LD Bio^®^: 21 of them presented apparent bands at 30 kDa, 31 kDa, and 40 kDa. The remaining two samples presented apparent bands at 30 kDa, 31 kDa, 33 kDa, and 40 kDa. The Architect^®^ Toxo IgG values for these sera ranged from 3.2 to 30.4 IU/mL, the median was 6.6 IU/mL with an interquartile range of 10.1 IU/mL. Moreover, 58 samples (71.6%) presented a negative LDBio^®^ test and were considered true false-positive Architect^®^ IgG results. As for the results of the *recom*Line^®^ immunoblots, there was no specific result according to age or gender.

### Analysis of the combined immunoblots results

**Figure 2:**
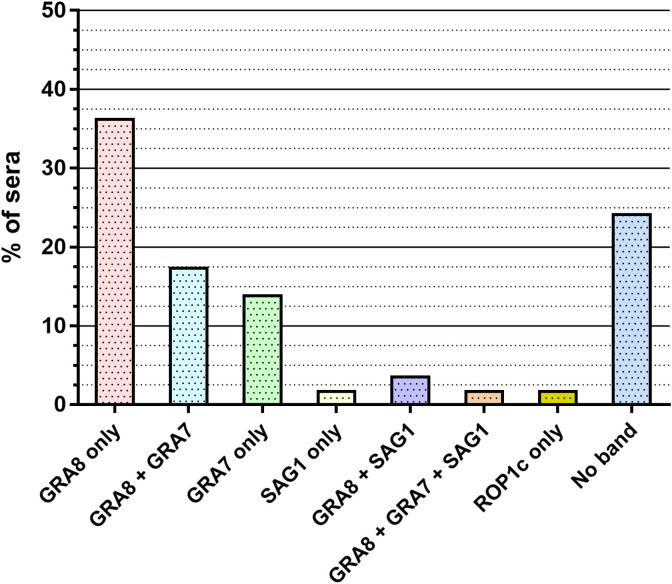
Description of the *recom*Line^®^ immunoblot profiles (for the 58 negative LDBio^®^ tests considered true false-positive sera with Architect^®^).

**Figure 3:**
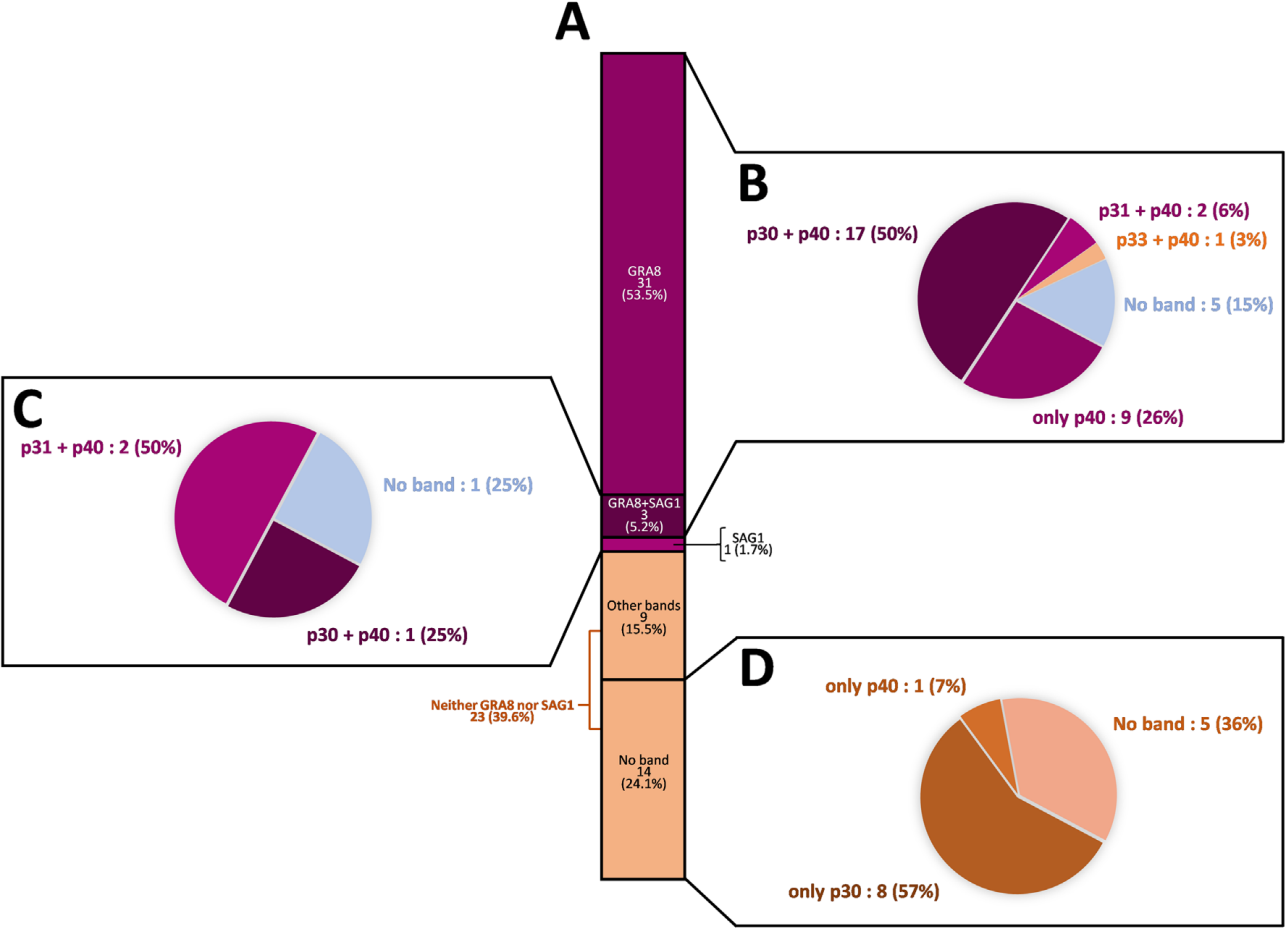
Description of the immunoblot profiles of the 58 negative LDBio^®^ tests (true false-positives with Architect^®^). (A) Proportion of *recom*Line^®^ profiles positive for GRA8, SAG1, GRA8+SAG1, other bands or no band, among the 58 negative LDBio^®^ tests. (B) LDBio^®^ profiles of the GRA8-positive *recom*Line^®^ profiles. (C) LDBio^®^ profiles of the SAG1-positive *recom*Line^®^ profiles. (D) LDBio^®^ profiles of the “no band” *recom*Line^®^ profiles.

The negative LDBio^®^ profiles are further detailed in Figures 2 and 3. Concerning these samples, *recom*Line^®^ immunoblots show that GRA8 (p35) and/or SAG1 (p30) appeared in 35 samples (60.4%) (including 31 profiles (53.5%) with GRA8, 1 (1.7%) with SAG1 and 3 (5.2%) with GRA8 + SAG1) (Figs. 2 and 3A). This may explain the false positivity with Architect^®^, which uses the recombinant antigens GRA8 and p30. On the other hand, 23 samples (39.6%) did not show either a GRA8 or p30 band and yet were positive with Architect^®^ (Figs. 2 and 3A). In detail, for these samples, *recom*Line^®^ patterns showed no band for 14 samples (24.1%), only GRA7 for 8 samples (13.8%), and only ROP1c for 1 sample (1.7%). For the samples containing the GRA8 band by *recom*Line^®^, the majority (50%) presented a p30 + p40 LDBio^®^ profile and 5 samples (15%) did not present any band by LDBio^®^ (Fig. 3B). Of the 4 samples containing the SAG1 band by *recom*Line^®^, 1 presented a p30 band by LDBio^®^ (Fig. 3C). Concerning the samples without any *recom*Line^®^ band, 36% also did not present any band by LDBio^®^, and 57% presented a p30 band (Fig. 3D).

For the 23 positive LDBio^®^ tests, all the samples presented the GRA8 band by *recom*Line^®^. Although all the positive LDBio^®^ tests had a p30 band by definition, 1 out of 23 presented the SAG1 band by *recom*Line^®^.

Among the 81 LDBio^®^ tests performed, 33 showed only the GRA8 band by *recom*Line^®^, of which 12 samples (36.4%) were positive and 21 (63.6%) were negative by LDBio^®^.

### Protein BLAST analysis

To assess the possibility of other cross-reactivities, we blasted protein sequences of *T. gondii* GRA8, GRA7, and SAG1 (p30) (Table 3). First, the *T. gondii* GRA8 protein showed high similarity to the *Hammondia hammondi* GRA8 protein (dense granule) in the Protein BLAST analysis. In the same way, the *T. gondii* GRA7 protein showed strong similarity with the *H. hammondi* GRA7 protein. Moreover, it also seems to have sequence homology with the uncharacterised protein NCLIV_021640 from the parasite *Neospora caninum*. On the other hand, there is no significant homology between *T. gondii* GRA7 and GRA8 proteins. Concerning *T. gondii* p30, its sequence homology with *N. caninum* surface protein P36 is substantial.

**Table 3:** Protein BLAST analysis.

Blasted proteins	Bitscore	*E*-value	Query cover (%)	Identities (%)	Positives (%)	Gaps (%)
*T. gondii* GRA8 vs. *H. hammondi* GRA8	275.0	2e–89	100	75	82	1
*T. gondii* GRA7 vs. *H. hammondi* GRA7	260.0	4e–84	99	60	70	2
*T. gondii* GRA8 vs. *T. gondii* GRA7	12.7	9.1	4	50	58	41
*T. gondii* GRA7 vs. *N. caninum* 021640	68.6	3e–10	91	36	47	21
*T. gondii* p30 vs. *N. caninum* P36	317.0	5e–104	94	52	66	1

## Discussion

All serological methods can provide false-positive results, irrespective of the infectious disease tested. The Architect^®^ Toxo IgG assay is currently one of the most commonly used for *T. gondii* serological diagnosis in France. In this study, we collected all suspected false-positive Architect^®^ IgG test results, regardless of the gender. Despite proven high specificity, the few false-positive test results are still a serious issue, particularly for pregnant women. In immunocompromised patients, reactivation of a past infection can be fatal, mainly from brain damage. In this specific population, a false-positive IgG test result can be misleading and appear to indicate cerebral toxoplasmosis, thus neglecting other differential diagnoses (e.g., cryptococcal meningitis or primary central nervous system lymphoma). In pregnant women, acute infection or reactivation of *T. gondii* can lead to a fatal outcome for the foetus. Importantly, a false-positive IgG test result in a pregnant woman can lead to stopping preventive measures, leading to the risk of an acute infection during pregnancy. In addition, false-positive IgG test results will make it difficult to diagnose an acute infection as it will be more difficult to differentiate the false-positive IgG from the neosynthesized ones of the acute infection. In such a situation, testing IgM, IgA, and avidity will be of utmost importance for the diagnosis. On the basis of our results, it seems that Architect^®^ IgG false positivity may be due to reactivity against GRA8 for the majority of the sera and GRA7 to a lesser extent. We also noted that a significant part of our samples did not present reactivity against any of the seven different recombinant proteins loaded on the *recom*Line^®^ immunoblot.

Previous studies on *T. gondii* cross-reactivity and the BLAST analyses performed in this work have led us to take an interest in the protozoan *H. hammondi*. *Hammondia hammondi* is another obligate intracellular parasite that infects cats. It is closely relative to *T. gondii* in terms of morphology, biology and genetics [19, 35]; however, this parasite is not known to be infective in humans [45]. Its range of intermediate hosts seems to be more restrictive and includes mainly rodents [16]. *Hammondia hammondi* also produces a GRA8 protein (dense granule) so the hypothesis of cross-reactivity with some of its antigens should be assessed.

Given the BLAST analysis results, it would be interesting to test our samples for *N. caninum* too. This parasite, like *H. hammondi*, is not known to be infective in humans and is found in dogs and other mammals. As remarkably reviewed by Gondim et al., several studies in the past forty years have shown serological cross-reactivities and cross-immunity between *T. gondii*, *H. hammondi*, and *N. caninum* [19]. *In vivo*, rodents infected with *H. hammondi* developed immunity and were protected against *T. gondii* lethal dose infection [15]. It is interesting to note that the immunogenic potential seems to depend on the strains used for experiments [3]. Immunisation with *H. hammondi* protects goats from abortion induced by *T. gondii* [31]. Serological cross-reactivities have also been found between these two parasites in mice, rabbits, dogs, and pigs using immunofluorescence, haemagglutination, dye-test or ELISA [19]. In another study reviewed by Gondim et al., five *T. gondii* antigens of molecular weight 30, 32, 35, 66, and 90 kDa were recognised using polyclonal anti-*H. hammondi* serum [35].

Concerning *N. caninum*, Gondim et al. reviewed studies showing that cross-immunity and protection of mice against *T. gondii*, after immunisation by *N. caninum*, are dependent on strains and doses used for experimental infections [19]. Serological cross-reactivities with *T. gondii* have also been a problem for the production of monoclonal antibodies against *N. caninum* [28, 39]. These findings highlight the fact that the strains of pathogens such as *H. hammondi*, *T. gondii*, and *N. caninum* are important in terms of cross-reactivity issues.

Moreover, the number of *recom*Line^®^ immunoblots without any positive bands raises additional questions. Despite the fact that none of them had a positive LDBio^®^ test (defined by three bands including p30), some nonetheless showed a p30 band not found by *recom*Line^®^. This inconsistency from one assay to another could be due to a difference of sensitivity between these two immunoblots. The LDBio^®^ strips are indeed coated with natural *T. gondii* antigens, whereas *recom*Line^®^ uses recombinant proteins.

Our results show that several negative serological tests do not guarantee true IgG negativity of the sera, given the positivity of 28.4% of the tested samples with the LDBio Toxo II IgG^®^ confirmatory test. The main assumption that could explain this result is that individual variations in the quantity of circulating IgG are high after *T. gondii* infection. Very low titres could point out the lack of sensitivity of the automated assays compared to the LDBio^®^ test facing such antibody titres.

As a reminder, the Architect^®^ automated assay is based on the immune reactivity of the sera against the proteins p30 and GRA8. The discrepancies we found between the positivity with Architect^®^ and yet the absence of GRA8 or p30 bands in immunoblots make us wonder whether the antigens chosen for automated assays are the most suitable ones. Most manufacturers do not in fact indicate what antigens are used in their test, and this can also be entire *T. gondii* antigenic extracts. A comparison between the different assays is then difficult to perform. We might wonder why the GRA8 antigen was initially added to the Architect^®^ IgG assay. Was the aim to increase sensitivity in the detection of past *T. gondii* infection or to allow earlier diagnosis of recent infection? Some previous studies seems to show that the recombinant GRA8 antigen allows for better diagnosis of acute toxoplasmosis rather than chronic infection [11, 21]. Moreover, we can question whether the antibodies directed only against GRA8, as we highlighted in our study in 46.6% of samples, are sufficient to consider the patient immunised against *T. gondii*? In this regard, among the *recom*Line^®^ tests only positive for GRA8 and tested by LDBio^®^, we found 36.4% positive and 63.6% negative confirmatory tests. Thus, in our sample set, some antibodies directed only against GRA8 could be considered true positive. The research is ongoing to find new efficient antigens. Innovative tools like bioinformatic analyses or epitope mapping have been developed and recent studies used chimeric antigens and multiepitope peptides for the diagnosis of acute and chronic infections [8, 10, 36]. Promising results in terms of sensitivity and specificity are available with these antigens, but more tests are needed to implement them in routine diagnostic practice [7, 20].

Since the selection of antigens for immunoassays remains an issue, the question becomes whether serological methods could be supplemented by other tests for the diagnosis of *Toxoplasma* infection. Humoral response of the host to the infection increases levels of circulating anti-*Toxoplasma* immunoglobulins. The different isotypes IgG, IgM, IgA, and IgE are currently used for diagnosis and estimation of the date of infection in serological tests [8, 38]. However, immune response to *T. gondii* is processed for a significant part by cell-mediated immunity [9, 12]. The first mechanism involved is a T-cell-independent response. *T. gondii* activates microbicidal functions of macrophages and synthesis of gamma interferon (IFN-γ) by natural killer cells [9]. The second mechanism involves interleukin-12 release by the macrophages, in response to the infection, to allow synthesis of IFN-γ by *Toxoplasma*-specific CD4^+^ and CD8^+^ T-cells through a Th1 immune response [9, 12]. IFN-γ is thus important to control parasite replication during the acute and chronic phases of the infection.

New tools based on cell-mediated immunity should be developed in order to be used in routine laboratories for the diagnosis of *T. gondii* infection. First, fluorescence-activated cell sorting (FACS) methods have been developed to detect patient-specific T-cell activation in blood after *in vitro* incubation with *T. gondii* antigens [13, 23]. More recently, the *in vitro* IFN-γ production of patient T lymphocytes after contact with *Toxoplasma* antigens has been assessed in a new test based on the principle of the IFN-γ assay, well described for the diagnosis of tuberculosis [1, 2, 5]. These tests were first designed to improve the diagnosis of congenital toxoplasmosis in infants born to mothers who seroconverted during pregnancy. Serological diagnosis of such patients is difficult since maternal *Toxoplasma*-specific IgG can cross the placenta, whereas the *Toxoplasma* IFN-γ assay allows for direct assessment of newborn cell-mediated immunity. In addition, this test also showed very good performances on adult patients [1].

Altogether, our work shows that the GRA8 and GRA7 proteins seem to be an avenue worth exploring to explain serological cross-reactivities. It highlights the importance of always confirming IgG positivity with at least another assay. False-positive samples cannot yet be avoided, even though they remain rare, and particular attention must be given to pregnant women with no proven past *T. gondii* infection. Serological tests have perhaps reached their limits and innovative tools such as cell-mediated immunity-based assays could become a valuable aid for toxoplasmosis diagnosis in the near future.

## Supplementary Materials

Supplementary material is available at https://www.parasite-journal.org/10.1051/parasite/2020006/olm Supplementary Table 1. Detailed results of all tests performed.

## Conflict of interest

The authors declare that Abbott Laboratories provided the *recom*Line^®^ kits used to perform part of the analyses.
